# Chaotic Map with No Fixed Points: Entropy, Implementation and Control

**DOI:** 10.3390/e21030279

**Published:** 2019-03-14

**Authors:** Van Van Huynh, Adel Ouannas, Xiong Wang, Viet-Thanh Pham, Xuan Quynh Nguyen, Fawaz E. Alsaadi

**Affiliations:** 1 Modeling Evolutionary Algorithms Simulation and Artificial Intelligence, Faculty of Electrical and Electronics Engineering, Ton Duc Thang University, Ho Chi Minh City, Vietnam; 2 Department of Mathematics and Computer Science, University of Larbi Tebessi, Tebessa 12002, Algeria; 3 Institute for Advanced Study, Shenzhen University, Shenzhen 518060, China; 4 Nonlinear Systems and Applications, Faculty of Electrical and Electronics Engineering, Ton Duc Thang University, Ho Chi Minh City, Vietnam; 5 National Council for Science and Technology Policy, Hanoi, Vietnam; 6 Department of Information Technology, Faculty of Computing and IT, King Abdulaziz University, Jeddah 21589, Saudi Arabia

**Keywords:** chaotic map, fixed point, chaos, approximate entropy, implementation

## Abstract

A map without equilibrium has been proposed and studied in this paper. The proposed map has no fixed point and exhibits chaos. We have investigated its dynamics and shown its chaotic behavior using tools such as return map, bifurcation diagram and Lyapunov exponents’ diagram. Entropy of this new map has been calculated. Using an open micro-controller platform, the map is implemented, and experimental observation is presented. In addition, two control schemes have been proposed to stabilize and synchronize the chaotic map.

## 1. Introduction

Discrete maps have attracted significant attention in the study of dynamical systems [1,2,3,4]. Discrete maps appear in various disciplines including physiology, chemistry, physics, ecology, social sciences and engineering [3,5,6,7]. It has previously been observed that simple first-order nonlinear maps can generate complex dynamical behavior including chaos [8]. Chaotic maps such as Hénon map [9], Logistic map [8], Lozi map [10], and zigzag map [11] are found. When investigating chaotic maps, the stability of fixed point plays a vital role. The authors tried to find fixed points and studied the behavior of orbits near fixed points. Relation of fixed points and critical transitions is illustrated in [12]. Previous studies have established that conventional chaotic maps often have unstable fixed points.

More recent studies have focused on chaotic maps related to the hidden attractor category [13,14,15]. Hidden attractors in chaotic maps are reported in [16], where a 1D map with no fixed point is extended from Logistic map. Jiang et al. introduced a list of two-dimensional maps with no fixed point [17]. These maps are inspired by Hénon map. By applying a Jerk-like structure, a gallery of 3D maps having hidden dynamics is investigated [17]. Ouannas proposed a fractional map having no fixed point [18]. Xu et al. found hidden dynamics of a two-dimensional map based on Arnold’s cat map [19]. The authors built a hardware implementation of the map using Field-programmable gate array (FPGA). However, detailed investigation of chaotic maps without fixed point should be examined further.

Our work discovers a new no equilibrium map with chaos. In Section 2, the map’s model is introduced, and its dynamics is reported. Realization of the map in an Arduino Uno board is presented in Section 3. In Section 4, control approaches for such a map are designed. Section 5 summarizes our work.

## 2. Chaotic Map

By using nonlinear functions, we construct a map described by:

(1) $$\left\{\begin{array}{c} x\left(n+1\right)=x\left(n\right)+y\left(n\right),\\ y\left(n+1\right)=y\left(n\right)-a\left|y\left(n\right)\right|-x\left(n\right)y\left(n\right)+b{\left(x\left(n\right)\right)}^{2}-c{\left(y\left(n\right)\right)}^{2}+d, \end{array}\right.$$

where *a*, *b*, *c*, and *d* are positive parameters.

The fixed points E(x,y) of the map can be found by solving

(2) $$\left\{\begin{array}{c} x=x+y,\\ y=y-a\left|y\right|-xy+bx^{2}-cy^{2}+d. \end{array}\right.$$

By rewriting Equation (2), we have

(3) $$bx^{2}+d=0.$$

Therefore, there is no any fixed point in the map in Equation (1) for such positive parameters *b* and *d*.

**Figure 1:**
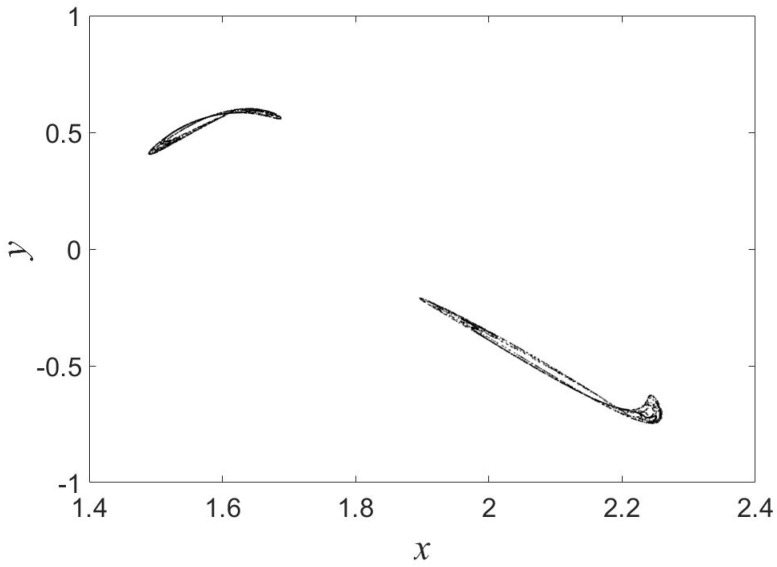
Strange attractor of the map for a=0.01, b=0.1, c=2, d=0.1 and (x(0),y(0))=(1.5,0.5).

We observe chaos in the map for a=0.01, b=0.1, c=2, d=0.1 and the initial conditions (x(0),y(0))=(1.5,0.5) (see Figure 1). Similar to the reported map in [18], the map in Equation (1) belongs to a class of maps without fixed point. Compared with the map reported in [18], the map in Equation (1) is not a fractional one.

### 2.1. Dynamics of the Map

**Figure 2:**
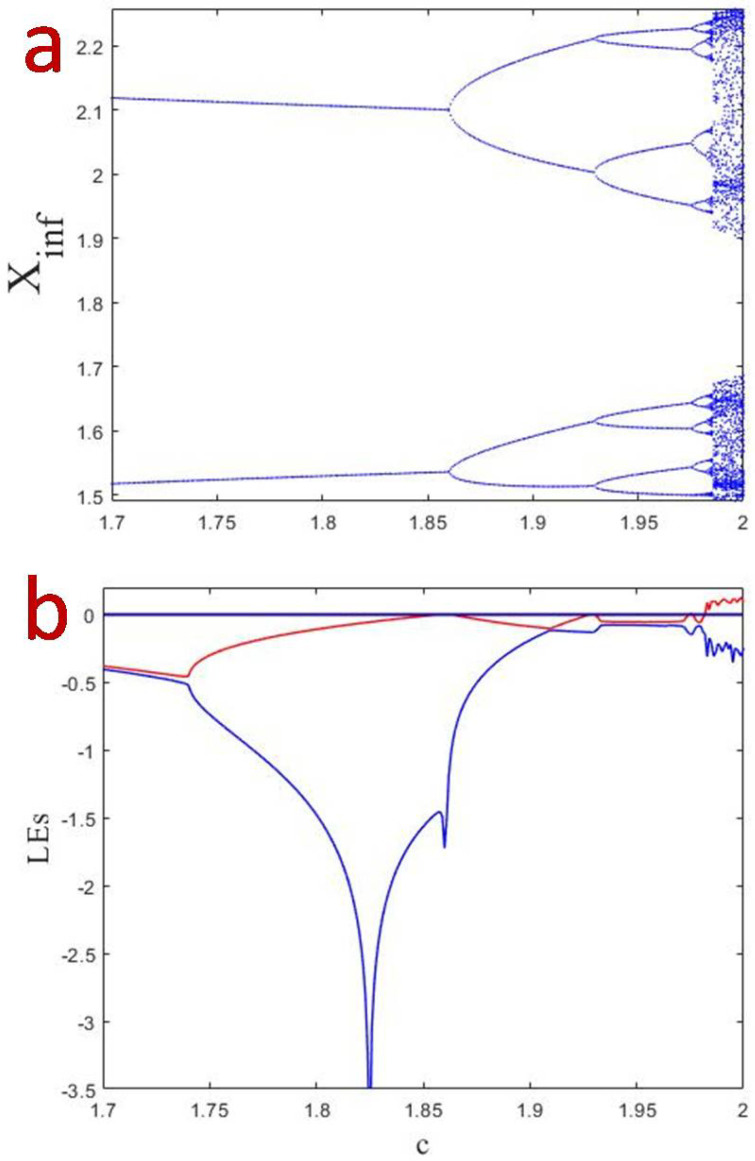
Bifurcation diagram (**a**); and Lyapunov exponents (**b**) when varying *c* for a=0.01, b=0.1, d=0.1 and (x(0),y(0))=(1.5,0.5).

Dynamics of the proposed map were studied. It was found that the map displays interesting dynamics when varying the parameter *c* and keeping a=0.01, b=0.1, d=0.1 and (x(0),y(0))=(1.5,0.5). Note that, since we wanted to keep the system NE (no equilibrium), we have frizzed the parameters *b* and *d*. Changing parameter *a* as bifurcation parameter did not show a proper route to chaos and in some values resulted in unbounded solutions. Thus, we chose *c* as the bifurcation parameter. In addition, note that the initial condition used in our simulations was not dominant and affected only the initial transient regime. As seen in the bifurcation diagram (Figure 2a) and the finite-time local Lyapunov exponents (Figure 2b), the map in Equation (1) displays a period doubling route to chaos. The time interval for calculating finite-time local Lyapunov exponents [20] is 10,000. Since it has no equilibrium, it has no period-1 cycle. The bifurcation starts from a period-2 cycle. Then, it continues with period-doubling until chaos is born a little before c=2.

### 2.2. Entropy of the New Map

Previous research has established that entropy is an effective index for estimating information in a particular system [21,22,23]. The authors applied entropy measurement to consider the complexity/chaos of dynamical systems [24,25,26,27]. In particular, approximate entropy (ApEn) [28,29] is useful to study chaotic systems [19,30]. It is noted that there is no reported threshold to be achieve in the ApEn in order to exhibit chaos [28,29]. Xu et al. reported the ApEn of a new system with chaos [19]. Their values of ApEn ranged from 0 to 0.12. Wang and Ding presented a table of AnEn test for four chaotic maps [30]. Here, calculated approximate entropy (ApEn) for the proposed the map in Equation (1) is reported in Table 1. Obtained entropy in Table 1 illustrates the complexity of the map when it exhibits chaos.

## 3. Implementation of the Map Using Microcontroller

**Figure 3:**
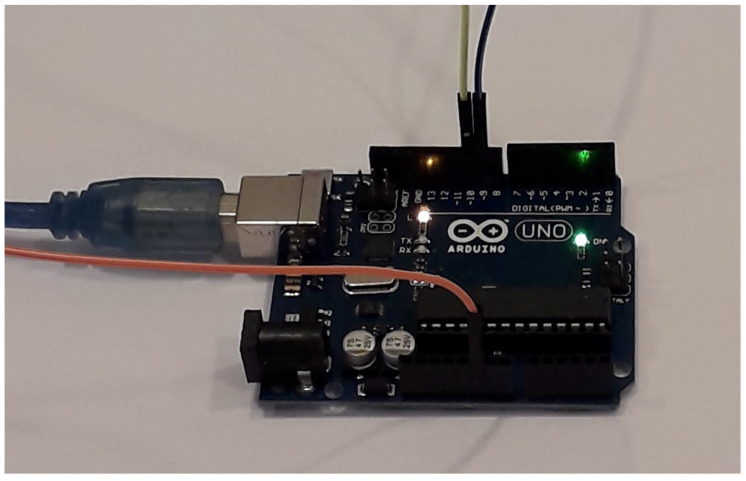
Arduino Uno board for implementing chaotic the map in Equation (1).

**Figure 4:**
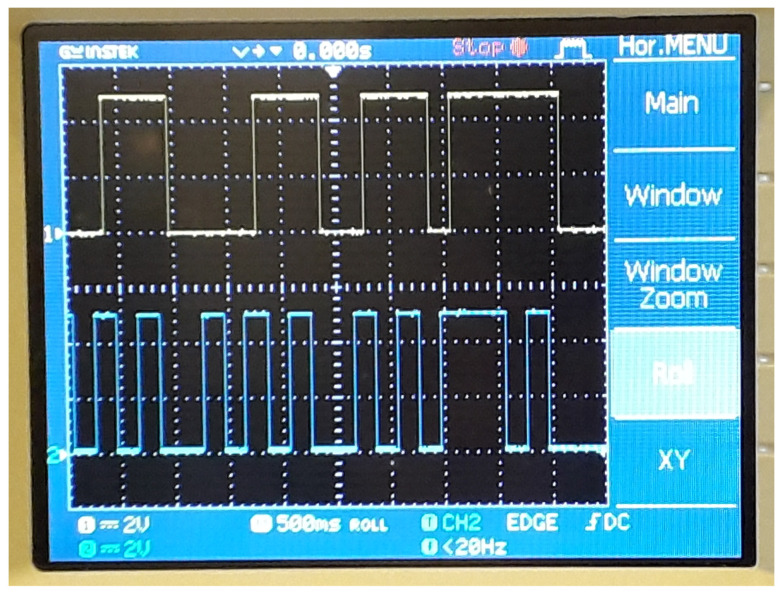
Captured waveforms at pins 9 and 10 of the Arduino Uno board.

Chaotic maps are useful for designing pseudorandom number generators [31,32,33,34], building S-Box [35], proposing color image encryption [36] or constructing secure communication [37]. Therefore, implementation of chaotic maps is a practical topic in the literature. Some approaches have been used to realize theoretical models of chaotic maps. Valtierra et al employed a skew-tent map in switched-capacitor circuits [6]. Bernoulli shift map, Borujeni maps, zigzag, and tent are done with a field-programmable gate array architecture [7]. Wang and Ding introduced FPGA hardware implementation of a map with hidden attractors [30]. It is worth noting that using microcontroller is an effective approach to implement chaotic maps [37,38]. The open-source platform named Arduino provides a reasonable development tool because of its free development software [39,40,41]. In our work, we used an Arduino Uno board based on microcontroller to realize the proposed map in Equation (1), as shown in Figure 3. Pins 9 and 10 of the Arduino Uno board are configured as two digital outputs. However, we could choose different pins for digital outputs because Arduino Uno board has 14 digital pins. We wrote a program for the map in the Arduino development environment. It is noted that the algorithm steps and program structure used in our implementation are similar to those reported in [38]. The output pin 9 was activated when x>1.8 while the output pin 10 was activated when y>0. Figure 4 displays the experimental waveforms at pins 9 and 10.

## 4. Control Schemes for the Proposed Map

When investigating chaotic maps, stabilization and synchronization are vital aspects. Two control laws for stabilizing and synchronizing the proposed non-fixed-point map are introduced in this section.

### 4.1. Stabilization

The aim of stabilizing the proposed map is to devise an adaptive control law such that all system states are stabilized to 0. The controlled map is

(4) $$\left\{\begin{array}{c} x\left(n+1\right)=x\left(n\right)+y\left(n\right)+u_{x},\\ y\left(n+1\right)=y\left(n\right)-a\left|y\left(n\right)\right|-x\left(n\right)y\left(n\right)+bx^{2}\left(n\right)-cy^{2}\left(n\right)+d+u_{y}, \end{array}\right.$$

where $u_{x}$ and $u_{y}$ are controllers to be determined.

The map in Equation (4) can be stabilized with the control law in Equation (5)

(5) $$\left\{\begin{array}{c} u_{x}=-\frac{1}{2}x\left(n\right),\\ u_{y}=-\frac{1}{2}y\left(n\right)+a\left|y\left(n\right)\right|+x\left(n\right)y\left(n\right)-bx^{2}\left(n\right)+cy^{2}\left(n\right)-d \end{array}\right.$$

Substituting the control law in Equation (5) into Equation (4), we get

(6) $$\left\{\begin{array}{c} x\left(n+1\right)=\frac{1}{2}x\left(n\right)+y\left(n\right),\\ y\left(n+1\right)=\frac{1}{2}y\left(n\right). \end{array}\right.$$

The written form of the error system in Equation (6) is

(7) $${\left(x\left(n+1\right),y\left(n+1\right)\right)}^{T}=M\times {\left(x\left(n\right),y\left(n\right)\right)}^{T},$$

where

(8) $$M=\left(\begin{array}{cc} \frac{1}{2} & 1\\ 0 & \frac{1}{2} \end{array}\right).$$

Therefore, the map in Equation (1) is stabilized.

**Figure 5:**
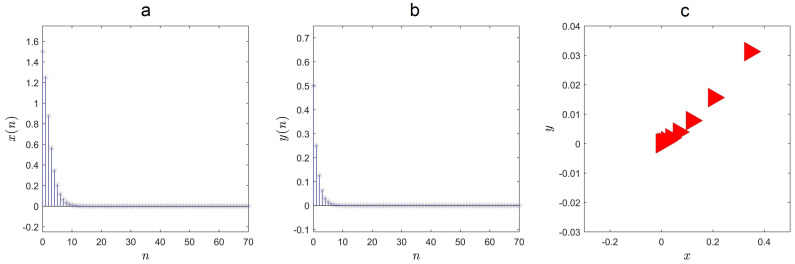
Stabilization when applying the proposed control law: (**a**) x(n), (**b**) y(n), and (**c**) x−y plane.

We illustrated the result by selecting parameters $\left(a,b,c,d\right)=\left(0.01,0.1,2,0.1\right)$ and $\left(x(0),y(0)\right)=\left(1.5,0.5\right)$. In Figure 5, the evolution of states verifies the control law.

### 4.2. Synchronization

Researchers have discovered synchronization of discrete systems [42,43,44]. We consider the drive system in Equation (9)

(9) $$\left\{\begin{array}{c} x_{m}\left(n+1\right)=y_{m}\left(n\right),\\ y_{m}\left(n+1\right)=x_{m}\left(n\right)+a_{1}x_{m}^{2}\left(n\right)+a_{2}y_{m}^{2}\left(n\right)-a_{3}x_{m}\left(n\right)y_{m}\left(n\right)-a_{4}, \end{array}\right.$$

It has been shown in [17] that the map in Equation (9) exhibits chaotic behaviors with no fixed points. The map in Equation (9) is one of the first example of discrete-time systems without fixed points, i.e, the map in Equation (9) has hidden attractors. The map in Equation (9) is inspired by the well-known Hénon map.

The subscript *s* denotes the response system’s states. The response is given by

(10) $$\left\{\begin{array}{c} x_{s}\left(n+1\right)=x_{s}\left(n\right)+y_{s}\left(n\right),\\ y_{s}\left(n+1\right)=y_{s}\left(n\right)-a\left|y_{s}\left(n\right)\right|-x_{s}\left(n\right)y_{s}\left(n\right)+bx_{s}^{2}\left(n\right)-cy_{s}^{2}\left(n\right)+d, \end{array}\right.$$

where $u_{i}\left(t\right)$(i=1,2) are synchronization controllers.

The error system is

(11) $$\begin{array}{ccc} e_{1}\left(n\right) & = & x_{s}\left(n\right)-x_{m}\left(n\right),\\ e_{2}\left(n\right) & = & y_{s}\left(n\right)-y_{m}\left(n\right), \end{array}$$

We find the controllers $u_{1}$ and $u_{2}$ based on Theorem 1.

**Theorem** **1.** *By selecting*

(12) $$\left\{\begin{array}{c} u_{1}=-\frac{1}{2}x_{s}\left(n\right)-\frac{1}{2}x_{m}\left(n\right)-\frac{2}{3}y_{s}\left(n\right)+\frac{2}{3}y_{m}\left(n\right),\\ u_{2}=\frac{1}{3}x_{s}\left(n\right)-\frac{2}{3}x_{m}\left(n\right)-\frac{3}{2}y_{s}\left(n\right)+\frac{1}{2}y_{m}\left(n\right)\\ \;\;\;\;\;\;a\left|y_{s}\left(n\right)\right|+x_{s}\left(n\right)y_{s}\left(n\right)-bx_{s}^{2}\left(n\right)+cy_{s}^{2}\left(n\right)-d\\ \;\;\;\;\;\;+a_{1}x_{m}^{2}\left(n\right)+a_{2}y_{m}^{2}\left(n\right)-a_{3}x_{m}\left(n\right)y_{m}\left(n\right)-a_{4}, \end{array}\right.$$

*the drive system in Equation (9) and the response system in Equation (10) are synchronized.*

**Proof.**  The error system in Equation (11) is rewritten as

(13) $$\left\{\begin{array}{c} e_{1}\left(n+1\right)=x_{s}\left(n\right)+y_{s}\left(n\right)-y_{m}\left(n\right)+u_{1},\\ e_{2}\left(n+1\right)=y_{s}\left(n\right)-a\left|y_{s}\left(n\right)\right|-x_{s}\left(n\right)y_{s}\left(n\right)+bx_{s}^{2}\left(n\right)-cy_{s}^{2}\left(n\right)+d\\ \;\;\;\;\;\;\;\;\;\;\;\;\;\;\;\;-x_{m}\left(n\right)-a_{1}x_{m}^{2}\left(n\right)-a_{2}y_{m}^{2}\left(n\right)+a_{3}x_{m}\left(n\right)y_{m}\left(n\right)+a_{4}+u_{2}, \end{array}\right.$$

Substituting the control law in Equation (12) into Equation (13) yields the reduced dynamics

(14) $$\left\{\begin{array}{c} e_{1}\left(n+1\right)=\frac{1}{2}e_{1}\left(n\right)+\frac{1}{3}e_{2}\left(n\right),\\ e_{2}\left(n+1\right)=\frac{1}{3}e_{1}\left(n\right)-\frac{1}{2}e_{2}\left(n\right). \end{array}\right.$$

The Lyapunov function is $V\left(e_{1}\left(n\right),e_{2}\left(n\right)\right)=e_{1}^{2}\left(n\right)+e_{2}^{2}\left(n\right)$,

$$\begin{array}{ccc} \Delta V & = & V\left(e_{1}\left(n+1\right),e_{2}\left(n+1\right)\right)-V\left(e_{1}\left(n\right),e_{2}\left(n\right)\right)\\ & = & \frac{1}{4}e_{1}^{2}\left(n\right)+\frac{1}{3}e_{1}\left(n\right)e_{2}\left(n\right)+\frac{1}{9}e_{2}^{2}\left(n\right)\\ & & \frac{1}{4}e_{1}^{2}\left(n\right)-\frac{1}{3}e_{1}\left(n\right)e_{2}\left(n\right)+\frac{1}{9}e_{2}^{2}\left(n\right)-e_{1}^{2}\left(n\right)-e_{2}^{2}\left(n\right)\\ & = & -\frac{1}{2}e_{1}^{2}\left(n\right)-\frac{7}{9}e_{2}^{2}\left(n\right)<0. \end{array}$$

By means of Lyapunov stability theory, the maps in Equations (9) and (10) are synchronized. □

**Figure 6:**
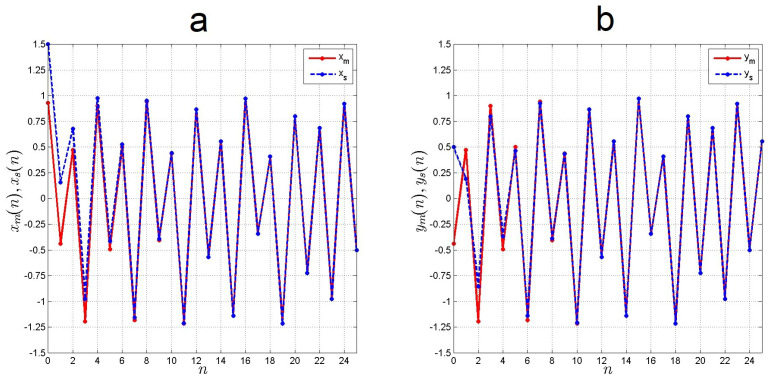
Evolution of states when applying the control: (**a**) $x_{m}\left(n\right)$, $x_{s}\left(n\right)$ and (**b**) $y_{m}\left(n\right)$, $y_{s}\left(n\right)$.

**Figure 7:**
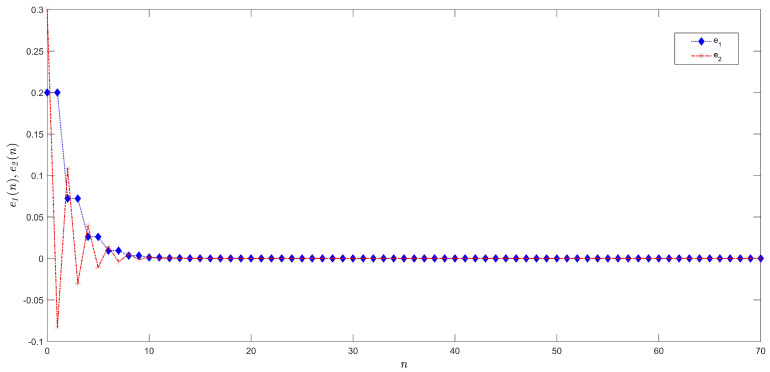
Synchronization errors.

Figure 6 depicts the time evolution of states of systems in Equations (9) and (10) after control. As reported in Figure 7, synchronization is obtained.

## 5. Conclusions

This work has introduced a new chaotic map, which can be considered as a system with hidden attractor. Having no fixed point is a notable feature of the proposed map. Chaos in the map is observed and confirmed by positive Lyapunov exponent. Realization of the map using an open-source electronic platform is given to illustrate its feasibility. Experimental results are recorded and displayed by oscilloscope. Approximate entropy is calculated to determine the complexity of the map. We have also presented stabilization and synchronization for the map. In future research, this map will be embedded into practical applications such as data encryption, signal transmission or motion planning.

## Figures and Tables

**Table 1:** Calculated approximate entropy of the map in Equation (1) for a=0.01, b=0.1, d=0.1 and (x(0),y(0))=(1.5,0.5).

Case	*c*	ApEn
1	1.985	0.0306
2	1.99	0.2142
3	1.995	0.2184
4	2	0.2525
